# Automated Categorization of Systemic Disease and Duration From Electronic Medical Record System Data Using Finite-State Machine Modeling: Prospective Validation Study

**DOI:** 10.2196/24490

**Published:** 2020-12-17

**Authors:** Gumpili Sai Prashanthi, Ayush Deva, Ranganath Vadapalli, Anthony Vipin Das

**Affiliations:** 1 Department of eyeSmart EMR & AEye LV Prasad Eye Institute Hyderabad, Telangana India; 2 International Institute of Information Technology Hyderabad , Telangana India

**Keywords:** electronic health records, data analysis, machine learning, algorithms, ophthalmology

## Abstract

**Background:**

One of the major challenges in the health care sector is that approximately 80% of generated data remains unstructured and unused. Since it is difficult to handle unstructured data from electronic medical record systems, it tends to be neglected for analyses in most hospitals and medical centers. Therefore, there is a need to analyze unstructured big data in health care systems so that we can optimally utilize and unearth all unexploited information from it.

**Objective:**

In this study, we aimed to extract a list of diseases and associated keywords along with the corresponding time durations from an indigenously developed electronic medical record system and describe the possibility of analytics from the acquired datasets.

**Methods:**

We propose a novel, finite-state machine to sequentially detect and cluster disease names from patients’ medical history. We defined 3 states in the finite-state machine and transition matrix, which depend on the identified keyword. In addition, we also defined a state-change action matrix, which is essentially an action associated with each transition. The dataset used in this study was obtained from an indigenously developed electronic medical record system called eyeSmart that was implemented across a large, multitier ophthalmology network in India. The dataset included patients’ past medical history and contained records of 10,000 distinct patients.

**Results:**

We extracted disease names and associated keywords by using the finite-state machine with an accuracy of 95%, sensitivity of 94.9%, and positive predictive value of 100%. For the extraction of the duration of disease, the machine’s accuracy was 93%, sensitivity was 92.9%, and the positive predictive value was 100%.

**Conclusions:**

We demonstrated that the finite-state machine we developed in this study can be used to accurately identify disease names, associated keywords, and time durations from a large cohort of patient records obtained using an electronic medical record system.

## Introduction

Electronic medical record (EMR) systems have been increasingly replacing paper-based records; using these systems has advantages such as increased efficiency and standardized quality, thereby enabling accurate clinical documentation [1]. Research that is dependent on reviewing paper records is not only cumbersome but also prone to human errors. The amount of time taken to retrieve and analyze large volumes of data from EMR systems is minimal compared to the manual process. Moreover, the adoption of EMR systems has led to the availability of diverse sources of clinical information, such as demographic data, history of diagnosis, prescriptions, and laboratory test results, which have established EMR systems as a treasure trove for large-scale analysis of health data. As a result, to obtain meaningful insights, there is a need to extract useful information and patterns from the rapidly growing volumes of data.

In general, 3 types of data are available as EMRs: structured, semistructured, and unstructured data [2]. Fixed-mode databases contain basic information and are usually used to store structured data. Unstructured data includes reports; records regarding surgery, medical history, and discharge; and clinical notes. One of the major challenges in the health care sector is that approximately 80% of the data remains unstructured and unused after it has been generated [3]. Since it is difficult to handle this sort of unstructured data obtained from EMRs, it tends to be neglected for analysis in most hospitals or medical centers [4]. Therefore, there is a need to analyze unstructured big data in health care systems so that we can optimally utilize the data and unearth all possible unexploited information from it.

The aim of this study was to extract a list of mentioned diseases and associated keywords, along with time durations, from the indigenously developed EMR system eyeSmart, which has been implemented across a large multitier ophthalmology network in India. We also aimed to describe the possibility of analytics from the datasets thus acquired.

## Methods

### Data Extraction

We retrieved systemic disease information of a subset of patients who presented to a large multitier ophthalmology network in India between August 2010 and December 2019 by using eyeSmart EMR system [1]. The dataset analyzed included the past medical history of patients and contained 10,000 records of distinct patients. From the given plaintext data about the medical history of the patients, we retrieved the names of systemic disease(s) from a fixed set of known disease names (Textbox 1) documented in the patients’ past medical history column and the duration of the disease, using Python and the techniques mentioned below.

**Search terms used to retrieve disease names and other associated keywords from the dataset.**

**Disease names (keyword) and their associated keywords:**

- Diabetes mellitus
  DM
  Insulin
  FBS
  PPBS
  IDDM
- Hypertension
  HTN
- Asthma
- Acid peptic disease
  Gastric
  Ulcer
- Hypothyroidism
- Hyperthyroidism
- Rheumatoid arthritis
  RA
- Allergy
- Tuberculosis
- Sinusitis
- Arthritis
  Joint pain
- Coronary artery disease
  CAD
- Cholesterol
- Migraine
- Cancer
- Paralysis
- Spondylitis
- Hepatitis
- Epilepsy
  Fits
  Seizures
- Malaria

### Data Availability

The dataset analyzed during the current study is not publicly available as it contains confidential patient information, but it can be made available from the corresponding author on reasonable request.

### Ethical Approval

This study was approved by the Institutional Review Board of LV Prasad Eye Institute, Hyderabad (Ethics Ref. No. LEC BHR-R-09-20-497), and all procedures were in accordance with the tenets of the Declaration of Helsinki. All data were fully anonymized prior to access by the study group.

### Assumptions About the Data

We made the following assumptions about the data in order to set some baselines for the information retrieval task:

1. The names of systemic diseases were spelled correctly.
2. Duration of the disease (if it exists) always followed the name of the disease and did not precede the documented disease.
3. If a disease name (D1) was followed by another disease name (D2) without any duration tag in between, then the duration for D1 was assumed to be missing and the next duration tag encountered would be associated with D2.

Given the unstructured plaintext data about systemic diseases and their durations, the following 2 steps were used to extract useful information and convert it into a structured data format.

#### Tag Identification

This involved the identification of disease names and duration tags in the extracted data. The task was to identify the presence of one or more of the enlisted diseases (Textbox 1) in the plaintext data. Since we assumed that the disease names were spelled correctly, we used string matching in Python to check if any disease names were present. Similarly, to identify the duration tags, we used regular expressions in Python to identify both (1) the value of duration (ie, a number) and (2) the unit of duration (ie, day, week, month, or year)

#### Clustering

This step involved correctly clustering the information, that is, finding and establishing the relations between different tags (in this case, duration and disease name).

Specifically, once the duration tags were identified, it was important to associate the correct duration with the corresponding disease, which was a challenging part.

Therefore, we propose a novel, finite-state machine (FSM) to sequentially detect and cluster disease name(s) from the patient’s medical history records.

**Figure 1:**
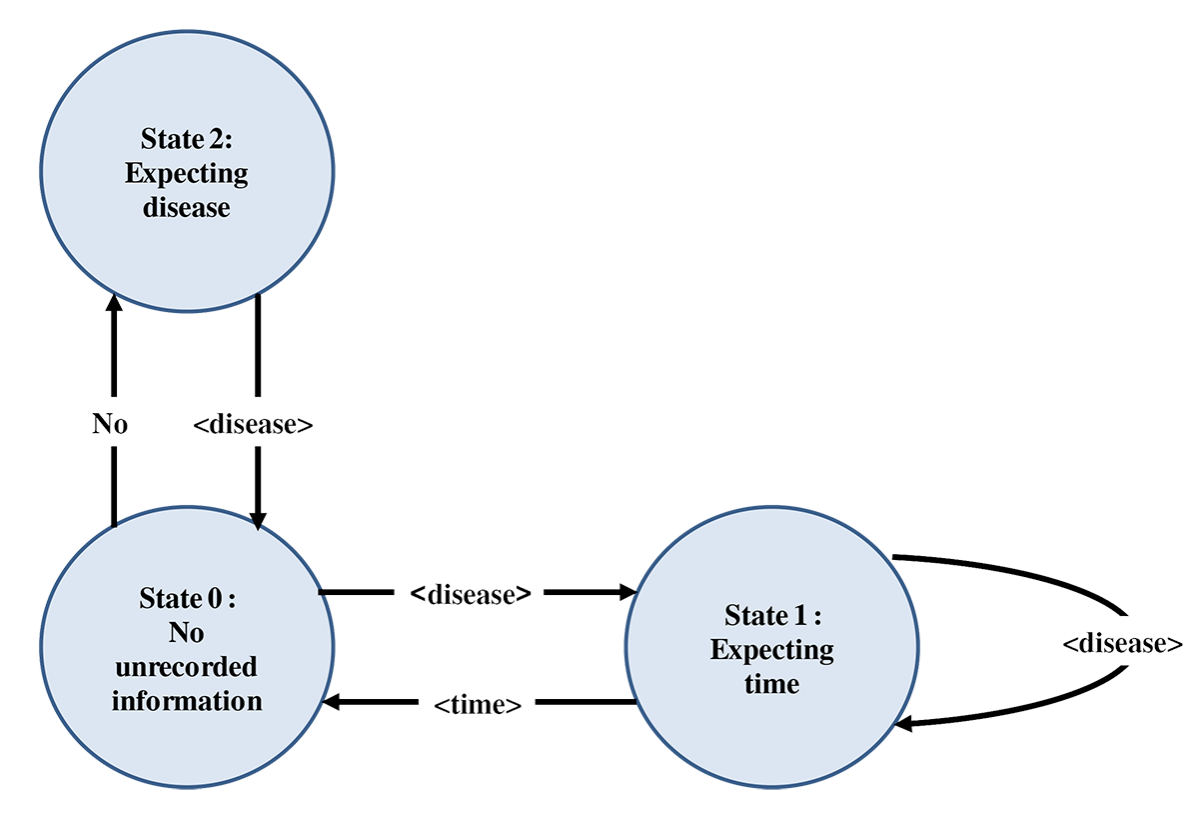
Flowchart depicting the 3 states of the finite-state machine modeling.

We defined 3 states in our FSM and the transition matrix that depends on the identified tag. In addition, we also defined a state-change action matrix, which is essentially an action associated with each transition. These are explained in detail below and illustrated in Figure 1.

### State Definition

#### State 0

This is the starting state. It has no unrecorded information. In this state, previous disease is “null” (prev_disease = NULL).

#### State 1

This is the state where we have found a disease previously that has not yet been recorded or stored in prev_disease. Here, we expect to find a time-matching regular expression to complete the record for that disease.

#### State 2

This is the state that is reached after encountering a “NO” string from State 0. A “NO” string indicates absence of the particular disease that follows the word “NO.” This means that the next sentence is about a disease that is not present and should not be included in the list. The state-change matrix and state-change action matrix are presented in Tables 1 and 2, respectively.

**Table 1:** State-change matrix.

Disease state	Input string		
	“NO”	Disease	Time
0	2	1	Error
1	Error	1	0
2	Error	0	Error

**Table 2:** State-change action matrix.

State	Input string		
	“NO”	Disease	Time
0	N/A^a^	1. Print(Found a disease. Hoping for it to be followed by a time since when disease existed.) 2. Update prev_disease = <disease>.	Print(Warning: Found a time unit before finding a disease)
1	Print(Disease Name followed by NO. Not according to how it should be)	1. Record <prev_disease, No time>. 2. Print(Warning: prev_disease was not followed by a time.) 3. Update prev_disease = <disease>	1. Record<prev_disease, <time>>. 2. Print(Successfully detected disease and time since when). 3. Update prev_disease = NULL.
2	Print(Error: Found two consecutive NOs)	Print(Detected a “NO Disease statement. Ignoring and not recording.)	Print(Found a time unit after NO. Something wrong)

^a^N/A: not applicable.

### FSM Output Measurements

We measured sensitivity, positive predictive value (PPV), and accuracy of the FSM to identify the disease name and associated keywords as well as the associated duration.

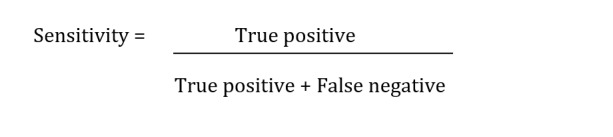

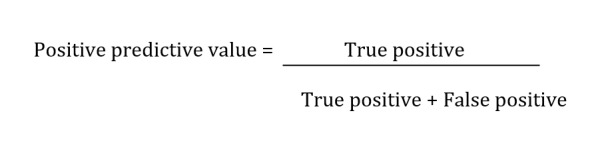

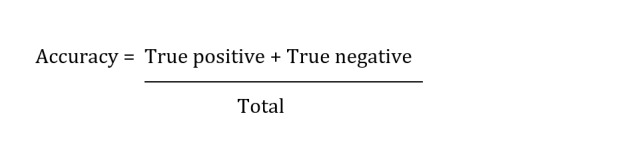







## Results

We evaluated the information extraction accuracy of the FSM by comparing the results to those of an expert human gold standard. The human had good knowledge about the medical terms used and their diagnoses. The report was further crosschecked by another person to minimize human error. In all, 100 records were randomly sampled and manually annotated and compared to the output of the algorithm. The record was annotated as true positive only when all the disease names and any associated keywords were accurately extracted along with the accurate disease duration.

To compare the predictions of the FSM to a gold standard (ie, manually annotated data, in our case), a confusion matrix was used. Table 3 represents a generic 2×2 confusion matrix used to identify the diagnosis. Table 4 represents a confusion matrix used to identify the duration associated with that particular diagnosis.

For the extraction of disease names and associated keywords, we reported an accuracy of 95%, sensitivity of 94.9%, and PPV of 100%. For the extraction of the disease duration, we reported an accuracy of 93%, sensitivity of 92.9%, and PPV of 100%.

**Table 3:** Confusion matrix to determine the diagnosis of disease (n=100).

Gold-standard method	FSM^a^ result	
	Predicted “Yes”	Predicted “No”
Actual “Yes”	94	5
Actual “No”	0	1

^a^FSM: finite-state machine.

**Table 4:** Confusion matrix to determine duration of the associated diagnosis (n=100).

Gold-standard method	FSM^a^ result	
	Predicted “Yes”	Predicted “No”
Actual “Yes”	92	7
Actual “No”	0	1

^a^FSM: finite-state machine.

## Discussion

### Principal Findings

In this study, we demonstrated that FSM can be used to accurately identify the disease name, associated keywords, and disease duration from a large cohort of patient records obtained using an EMR system that has been implemented across a large, multitier ophthalmology network in India. Many previous studies have used regular expressions and natural language processing (NLP) to extract disease names or keywords [5-17]. Hobbs et al [18] used cascading finite-state automatas for extracting information from natural language text [18]. Leroy et al [19] used finite-state automata to structure the relation between extract entities, but attempts to extract the duration of the disease along with the disease name itself have not been made previously.

A variety of valuable medical information is stored in texts that are unstructured, but there are many challenges in dealing with such data as the text may contain many errors, incorrect usage of grammar, and improper structural framework, which would increase the challenges in analyzing and processing of data.

Unstructured data gives a wider picture about patient data and aids clinicians in connecting the dots and presenting a more accurate picture of the health of the patient. Extracting useful information from these records help doctors to identify a patient’s medical history and also make important predictions.

There are inherent challenges in how information in an unstructured format is inputted into the EMR. This is governed by the training, literacy, and typing skill of the user in question. These challenges include errors in spelling and nonconformity of the structure of the data inputted. Automating the analysis of data in such formats helps reduce the time taken for manual mining of information.

### Comparison With Prior Work

In a study on asthma by Zeng et al [5], which involved using NLP for extracting principal diagnosis, comorbidity, and smoking status, the accuracy of the algorithm was 82%, 87%, and 90%, respectively. Rosier et al [6] used clinical records to extract data on pacemaker implantation procedures by using regular expressions. The system extracted information with a very high PPV (>95%) and sensitivity (>90%). In a study by Murtaugh et al [7], which involved extraction of body weight values from clinical notes, the accuracy was 98.3% and precision was 98.8%. These values are similar to our findings in this study involving identification of systemic diseases and their durations.

Systemic diseases are frequently considered to be the underlying cause of many medical conditions. Systemic disease history is a particularly important component in the examination of patients with eye disease. Various systemic diseases affect the eye, notably diabetic retinopathy, dry eye disease, cataract, and thyroid eye disease [20]. An understanding of the duration of the systemic diseases is vital to prognosticate the severity of the ocular condition and the treatment outcomes.

In a systemic review based on extracting information from the text of EMRs to improve case detection, Ford et al [8] compared the accuracy of case-detection algorithms by comparing codes and text. For codes-only algorithms, the median sensitivity was 61.7% and PPV was 72%. For text-only algorithms, the median sensitivity was 78.1% and PPV was 73%. Moreover, for a combination of text and codes, the median sensitivity was 78.1% and PPV was 86%. The medical conditions included in this review were respiratory infections, bowel disease, cancer, and diabetes. The algorithm sensitivity ranged from 48.4% to 99.2%, specificity ranged from 90% to 99.4%, and PPV ranged from 54% to 97.9% [8].

Zheng et al [13] used both structured and unstructured EMRs for developing and testing a web-based diabetes case detection algorithm. The NLP-based algorithm had a PPV of 90%. Petch et al [14] extracted 15 clinical features from dictated ambulatory consult notes by using a commercially available NLP-based tool. NLP performed best for features that were classified as simple, yielding an overall accuracy of 96%. However, the performance was lower for other features that were of moderate and complex linguistic complexity.

The scope of this study, as the first experiment of this nature, was to successfully categorize systemic diseases and their durations from a cohort of patient records. The next models will focus on categorizing clinical findings based on slit lamp examination of various parts of the eye and the plan of management written by the health care provider. The tasks we undertook in this study were relatively challenging. The major challenges were that a patient’s medical history may contain information about multiple diseases. The presence of the name of a disease does not always imply the patient was diagnosed with that disease. We can have instances where a doctor may write that the patient had no history of a particular disease. Moreover, not all disease names identified in the data had a duration associated with them. For example, there can be 3 disease names and only 2 duration related tags. All these challenges were addressed by the current methodology of FSM, as described in this study.

### Study Limitations

One of the limitations of this study is that if the duration of disease were preceded by the disease name, it could not be identified and that disease names could not be identified if there were any spelling mistakes.

Since the current dataset had negligible spelling mistakes, and the disease names were always followed by the duration, the state space of the current FSM was small. However, an advantage of modeling this as an FSM is that it can be easily extended to run on datasets where these assumptions do not hold true. Thus, we propose that FSM is a very robust framework to address challenges of automated systemic disease and duration categorization. Our findings also suggest that this method can be used more generally for both clinical and research purposes, to identify the disease and duration.

### Future Directions

Future work involves using the FSM on more datasets, understanding the complexities of the unstructured datasets that are used as inputs, and incorporating more changes to make the FSM more robust. This is an ongoing process of periodically analyzing the input data to modify the state changes to enable a more accurate categorization of the required variables.

The adoption of EMR in a large country like India is rather low. There are various associated challenges, but the potential long-term benefits for research and education are promising. Structured datasets from the EMR are crucial for any meaningful research to be conducted. Unstructured datasets also need to be analyzed in an automated fashion to minimize the time required for analyses.

### Conclusions

In conclusion, we present a novel technique that was developed to analyze unstructured data of systemic diseases and their durations in a large cohort of patient records in a multitier ophthalmology network in India.

## Abbreviations

EMR: electronic medical record
FSM: finite-state machine
NLP: natural language processing
PPV: positive predictive value
